# Altered Tissue and Plasma Levels of Fibroblast Activation Protein-α (FAP) in Renal Tumours

**DOI:** 10.3390/cancers12113393

**Published:** 2020-11-16

**Authors:** Jon Danel Solano-Iturri, Peio Errarte, María C. Etxezarraga, Enrique Echevarria, Javier Angulo, José I. López, Gorka Larrinaga

**Affiliations:** 1 Department of Anatomic Pathology, Donostia University Hospital, 20014 Donostia/San Sebastian, Spain; jondanel.solanoiturri@osakidetza.eus; 2 Department of Medical-Surgical Specialities, Faculty of Medicine and Nursing, University of the Basque Country (UPV/EHU), 48940 Leioa, Spain; 3 BioCruces Health Research Institute, 48903 Barakaldo, Spain; peio@onenameds.com (P.E.); mariacarmen.etxezarragazuluaga@osakidetza.eus (M.C.E.); joseignacio.lopez@osakidetza.eus (J.I.L.); 4 Department of Physiology, Faculty of Medicine and Nursing, University of the Basque Country (UPV/EHU), 48940 Leioa, Spain; enrique.etxebarria@ehu.eus; 5 Department of Anatomic Pathology, Basurto University Hospital, University of the Basque Country (UPV/EHU), 48013 Bilbao, Spain; 6 Department of Urology, University Hospital of Getafe, European University of Madrid, 28907 Madrid, Spain; javier.angulo@universidadeuropea.es; 7 Department of Anatomic Pathology, Cruces University Hospital, 48903 Barakaldo, Spain; 8 Department of Nursing, Faculty of Medicine and Nursing, University of the Basque Country (UPV/EHU), 48940 Leioa, Spain

**Keywords:** clear cell renal cell carcinoma, renal tumours, prognosis, FAP, cancer associated fibroblast

## Abstract

**Simple Summary:**

Malignant epithelial tumour’s behaviour in the kidney has traditionally been analysed attending to different prognostic parameters focussed on the proliferating neoplastic cell. This is the case of renal cell carcinoma (RCC), in which a large tumour diameter, high histological grade, and the presence of necrosis, among other factors, have been related with a high risk of distant metastasis and, therefore, worse survival. Recently, several elements of the tumour microenviroment, such as cancer-associated fibroblasts (CAFs), are being studied in order to develop more accurate diagnostic and therapeutic approaches. We present data that support that the fibroblast activation protein-α (FAP), a CAF biomarker, provides interesting information both in tumour tissues and in plasma from patients with RCC.

**Abstract:**

(1) Background: Renal cell carcinoma (RCC) is a heterogeneous and complex disease with only partial response to therapy, high incidence of metastasis and recurrences, and scarce reliable biomarkers indicative of progression and survival. Cancer-associated fibroblasts (CAFs) play an important role supporting and promoting renal cancer progression. (2) Methods: In this study, we analysed fibroblast activation protein-α (FAP) immunohistochemical expression and its soluble isoform (sFAP) in tumour tissues and plasma from 128 patients with renal tumours. (3) Results: FAP is expressed in the cell surface of CAFs of the tumour centre and infiltrating front from clear cell renal cell carcinomas (CCRCC, *n* = 89), papillary renal cell carcinomas (PRCC, *n* = 21), and chromophobe renal cell carcinomas (ChRCC, *n* = 8), but not in the benign tumour renal oncocytoma (RO, *n* = 10). A high expression of FAP and low levels sFAP are significantly associated with high tumour diameter, high grade, and high pT stage, lymph node invasion, development of early metastases, and worse 5-year cancer specific survival of CCRCC patients. (4) Conclusions: These findings corroborate the potential usefulness of FAP immunohistochemistry and plasma sFAP as a biomarker of CCRCC progression and point to CAF-related proteins as promising immunohistochemical biomarkers for the differential diagnosis of ChRCC and RO.

## 1. Introduction

Renal cell carcinoma (RCC) is the third most common genitourinary cancer and ranks within the top-ten list of the most frequent malignancies in Western countries [1,2]. Clear cell renal cell carcinoma (CCRCC) is the most common histological subtype (75–80%) and, together with papillary renal cell carcinoma (PRCC) (10–15%), it arises from the proximal tubule of the nephron [1,2]. The chromophobe renal cell carcinoma (ChRCC) (5% of cases) and the renal oncocytoma (RO) (5%), which is a benign tumour, are originated in the intercalated cells of the distal nephron and share a common histogenetic lineage [3]. 

The current state of the art in the diagnosis relies on the histologic findings and immunohistochemical profile of the tumours. Since radiotherapy and chemotherapy are inefficient therapies in renal tumours, only surgery has demonstrated a real impact in improving patients’ survival [3,4]. Then, RCC is far from being a single disease and research to discover new tumour markers of clinical significance is needed. Histologically defined variants follow different genetic drivers, variable therapeutic response to anti-angiogenic therapies, and pursue a different clinical evolution [5]. It is a classic observation that patients with CCRCC had a poorer prognosis compared with patients with PRCC and ChRCC [3]. However, systemic treatments (tyrosine kinase and immune checkpoint inhibitors) for patients with metastatic non-clear cell RCC tend to be significantly less effective than for CCRCC, and optimal therapy for non-clear cell RCC remains unclear and warrants further study [6].

Due to its phenotypic variability and intratumoural heterogeneity, CCRCC is considered to be one of the most challenging of human neoplasms [4]. Metastatic disease is present at diagnosis in around 30% of patients, and another third relapses and progresses to metastatic disease after nephrectomy [3]. The identification of alterations that may influence this unpredictable tumour behaviour and clinical outcome both in primary tumour tissues and in liquid biopsies is needed to improve the management of these patients [7]. With this regard, in recent years, novel prognostic biomarkers and therapeutic approaches based on tumour microenvironment have being explored. These include the use of immune checkpoint inhibitors, which is showing promising results for metastatic CCRCC [8]. In line with the strategy of targeting not only the neoplastic cells themselves, current attempts are being directed against other non-neoplastic cellular compartments, including cancer-associated fibroblasts (CAFs).

CAFs are a major constituent of tumour stroma, and they are even considered by some to be the architects of cancer [9], since the crosstalk between CAFs, cancer cells and other stromal cells is vital for the acquisition of several hallmarks of cancer. CAFs are known to participate in the epithelial to mesenchymal transition (EMT), as well as to contribute to the promotion of tumour growth and invasiveness, the escape from immunologic surveillance, and the remodelling of the extracellular matrix [10,11,12]. Regarding renal cancer, in vitro studies have demonstrated that the crosstalk communication between CAFs and tumour cells induces pro-invasive properties in RCC cell lines [12]. On the basis of these findings, CAF-associated biomarkers may well be promising diagnostic and therapeutic targets [10,11,12,13,14].

Fibroblast activation protein-α (FAP) is the universal biomarker of CAFs [15,16]. This multifunctional protein is found in epithelial cancers, both in tumour tissues and in body fluids [15,16,17,18]. Although no use of this biomarker in clinical routine has been protocolised yet, most of the immunohistochemical analyses described in different solid tumours show great potential as a prognostic marker since its expression is very significantly associated with tumour aggressiveness and worse survival [19]. Furthermore, the expression of this protein in CAFs of the tumour stroma, together with its absence from most normal tissues of the body [15,16], points to FAP as a very promising target not only for FAP-targeted therapies [14,15,16] but also for molecular imaging. Thus, it has been proposed very recently as the next “billion dollar” nuclear theranostic target [14]. First analyses in liquid biopsies have also demonstrated a prognostic potential for soluble FAP (sFAP) in several solid tumours [17,18,20].

The study of FAP in renal cancer was an unexplored territory until recent years. We analysed this protein in primary CCRCCs and in paired metastases [21,22]. FAP immunohistochemical expression was associated with high tumour diameter, tumour necrosis, sarcomatous phenotypes, high grade and high stage tumours, the development of early metastases, and worse survival of CCRCC patients. According with these results, The Cancer Genome Atlas (TCGA: https://www.cancer.gov/tcga) described that high mRNA expression of FAP is also significantly associated with worse overall survival [23]. These evidences lead us to hypothesise on the diagnostic/prognostic potential of this biomarker in tumour tissues but also in liquid biopsy of renal cancer patients. To shed light on this question, we set three main objectives for this study. The first objective was to validate previous results and to study the heterogeneity of FAP expression in a series of 89 CCRCCs, analysing it in both the central part and the infiltrating front of primary tumours. The second was to explore the correlation between tissue FAP expression and plasma levels of FAP from these 89 CCRCC patients, and to test preliminarily the prognostic impact of this soluble isoform of FAP (sFAP). The third objective was to study the expression of this protein in tissue and plasma samples from patients with papillary renal cell carcinomas (PRCC), chromophobe renal cell carcinomas (ChRCC), and renal oncocytomas (RO).

## 2. Results

### 2.1. Immunohistochemical (IHC) Expression and Plasma Levels of FAP According to Gender and Age of RCC Patients

The non-parametric Rho Spearman test was performed to assess if FAP protein expression and plasma levels vary according to the gender or age of the patients. Results showed no statistically significant differences in all cases, which allowed us to conclude that the sample has no gender or age bias (Table S1).

### 2.2. FAP Expression in Different Histological Subtypes of RCC and in Plasma Samples of Renal Tumour Patients and Control Subjects

**Figure 1:**
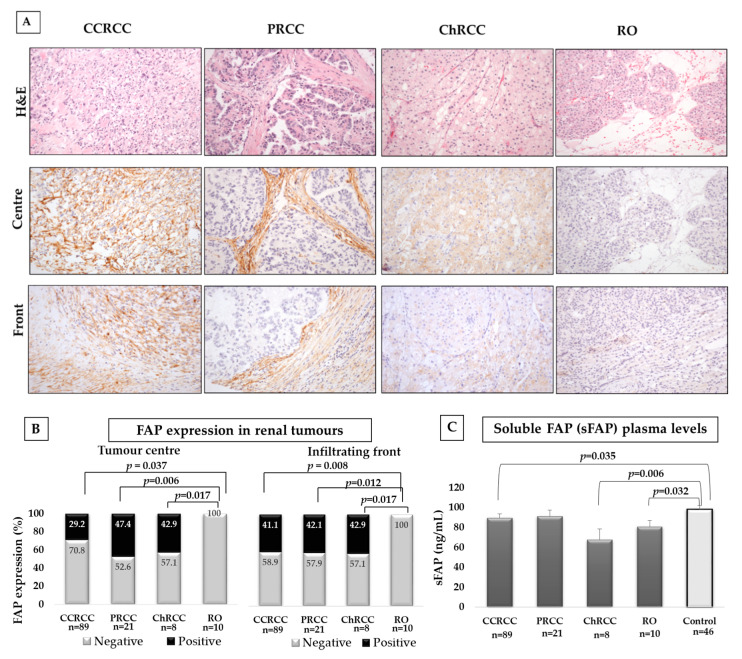
Immunohistochemical fibroblast activation protein-α (FAP) staining in renal tumours and plasma levels of soluble FAP (sFAP) in renal tumour patients and control subjects. (**A**) Upper row shows haematoxylin-eosin staining of renal tumours. Middle and lower rows show FAP immunostaining at the tumour centre and border, respectively. Original magnification, ×250. (**B**) Renal cell carcinomas (RCCs) expressed FAP, whereas renal oncocytoma (RO) was negative. Staining was scored as negative or positive. Chi-square test was performed for comparisons in tumour tissues. (**C**) Soluble FAP (sFAP) was significantly lower in plasma samples from CCRCC and ChRCC patients than in controls. A Mann–Whitney U-test was used for comparisons in plasma samples.

Figure 1A shows the differences of tissue FAP expression according to histological subtype. Renal cell carcinomas (CCRCC, PRCC, and ChRCC) showed similar percentages of FAP positive staining, whereas all the benign tumours (ROs) were negative. Significant differences were found between RCCs and RO both at the centre and at the infiltrating front (Figure 1B).

Due to the potential clinical relevance of the significant differences of FAP expression between ChRCC and RO [3], we performed Receiver Operating Characteristic (ROC) curves (Table S2). Since 100% of RO were negative for FAP and 42.9% of ChRCCs were positive, the specificity was of 100% and the sensitivity was of 42.9% (AUC = 0.714).

We also compared differences in the expression of FAP between tumour centre and border of RCCs. None of the three subtypes showed significant differences of FAP expression in both locations of tumours (CCRCC Chi-square test, *p* = 0.1; PRCC, *p* = 0.74; and ChRCC, *p* = 0.99). To deepen the analysis, we studied differences between the centre and border within CCRCCs with low or high histological grade, low or high pT, and within small or large CCRCCs. FAP expression in both tumour areas did not vary significantly in any case (Chi-square test, *p* > 0.05 in all cases) (data not shown in figures or tables).

Figure 1C shows plasma levels of sFAP in patients with renal tumours and in control subjects. sFAP was significantly lower in plasma samples from patients with renal tumours than in controls. However, we did not found any significant difference between plasmas from RCC patients and from patients with benign tumours (CCRCC vs. RO, Mann–Whitney U-test, *p* = 0.71; PRCC vs. RO, *p* = 0.27; ChRCC vs. RO, *p* = 0.15).

Finally, we compared plasma sFAP levels in terms of tissue FAP expression (Table 1). Thus, sFAP levels from patients with FAP negative RCCs were similar to patients with FAP positive tumours.

### 2.3. Tissue FAP Expression and sFAP Levels in Terms of CCRCC Aggressiveness

We stratified tissue FAP expression and plasma levels by clinical parameters tightly related to tumour aggressiveness such as Fuhrman histological grade, tumour size, local invasion (pT), presence/absence of affected lymph nodes (*N*), and time of presentation of distant metastasis (M).

#### 2.3.1. FAP and Fuhrman Grade

**Figure 2:**
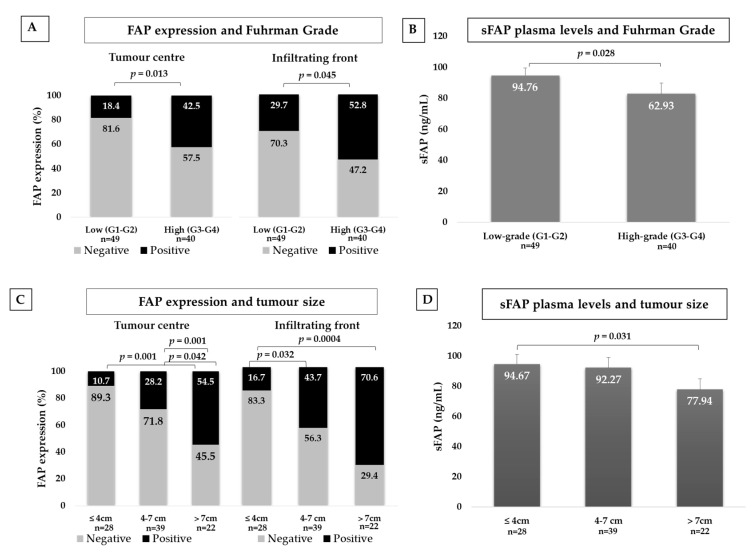
Immunohistochemical FAP staining and plasma levels of soluble FAP (sFAP) according to clear cell renal cell carcinomas (CCRCC) aggressiveness. FAP immunostaining in centre and border of tumours and plasma levels of CCRCC patients depending on histological grade (**A**,**B**) and tumour diameter (**C**,**D**). FAP staining intensity was scored as negative or positive (Chi-square test). Results in plasma samples were analysed with Mann–Whitney test.

The series only had three grade 1 CCRCCs; therefore, we stratified cases as low grade (G1–G2) and high grade (3–4). High-grade CCRCCs expressed higher percentages of FAP staining than low-grade tumours both at the centre and at the infiltrating front (Figure 2A). By contrast, plasma levels of sFAP were significantly lower in patients with high-grade CCRCCs (Figure 2B).

#### 2.3.2. FAP and Tumour Size

Results were stratified in three groups following previously reported classifications [24]: tumours with 4 cm or smaller, 4 to 7 cm, and larger than 7 cm. Thus, the larger the tumour, the higher the FAP positive staining, both at the centre and at the edge of the tumour (Figure 2C).

Soluble FAP levels showed an opposite pattern. Plasma from patients with CCRCCs larger than 7 cm showed lower levels than from patients with tumours smaller than 4 cm (Figure 2D).

#### 2.3.3. FAP and Local Invasion (pT)

**Figure 3:**
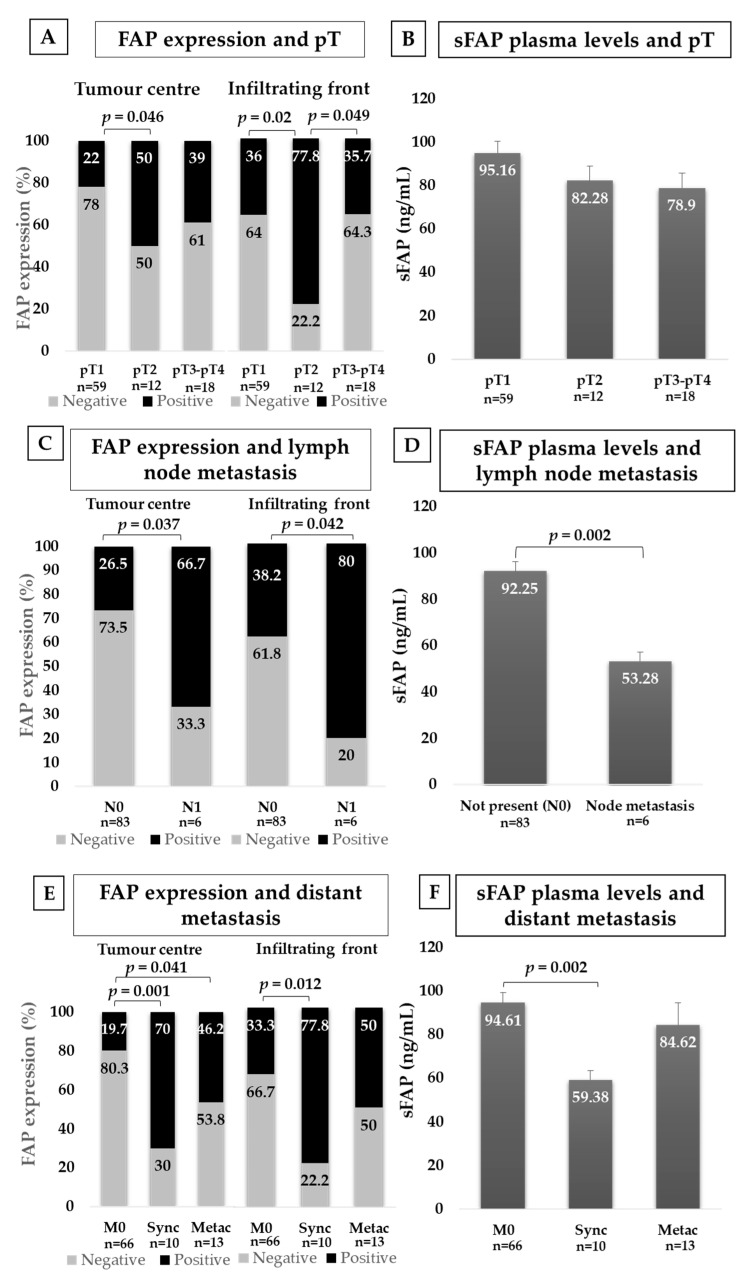
Immunohistochemical FAP staining and plasma levels of sFAP according to CCRCC aggressiveness. FAP immunostaining in centre and border of tumours and sFAP levels in plasma from CCRCC patients depending on local invasion (**A**,**B**), locoregional lymph node invasion (**C**,**D**) and distant metastasis (**E**,**F**). FAP staining intensity was scored as negative or positive and Chi-square test was used for comparisons. Results in plasma samples were analysed with Mann–Whitney U-test. N0: No lymph node metastasis; N1: lymph node metastasis; M0: No distant metastasis; Sync: Synchronous distant metastasis; Metac: Metachronous distant metastasis.

Due to the limited number of pT4 cases and taking into account the relevance of tumour size in the pT classification, we stratified local invasion into three groups: pT1 (organ confined tumours ≤ 7 cm), pT2 (organ-confined tumours > 7 cm) and pT3-pT4 (non-organ-confined tumours). FAP expression at the centre and at the edge of pT2 tumours significantly increased with respect to pT1 ones. We also observed higher expression at the infiltrating front of pT2 tumours when compared to non-organ-confined CCRCCs (Figure 3A).

Eight of the CCRCCs that invaded renal vein (pT3) were smaller than 7 cm. With the objective to better evaluate the influence of tumour size in the comparison with non-organ-confined tumours, we performed the same analysis after removing these eight cases (Figure S1). Thus, FAP-positive cases at the centre of the tumour were also significantly higher in non-organ-confined tumours and in pT2 tumours than in pT1 ones. At the infiltrating front of these non-organ-confined tumours, FAP-positive cases (62.5%) almost duplicated the expression of pT1 ones (36%), although it did not reach statistical significance (Chi-square test, *p* = 0.1). Differences were significant between pT2 and pT1 tumours (Figure S1A).

sFAP levels gradually decreased in plasma samples while local invasion increased, although this difference was not statistically significant (Kruskal–Wallis test, *p* = 0.13) (Figure 3B). Similar results were observed when tumours smaller than 7 cm were removed from the non-organ-confined pT3 group (Kruskal–Wallis test, *p* = 0.17) (Figure S1B).

#### 2.3.4. FAP and Lymph Node Invasion (*N*)

Tumours that invaded locorregional lymph nodes showed higher FAP expression, both at the tumour centre and border, than in tumours that did not invade (Figure 3C).

Plasma levels of sFAP were lower in patients with lymph node metastases than in patients without (Figure 3D).

#### 2.3.5. FAP and Distant Metastasis (M)

Following previously reported classifications [25], we stratified these results in three groups: (1) Tumours that did not metastasised during the follow-up of CCRCC patients, (2) Tumours that debuted with metastases within 6 months of the first primary cancer (synchronous), and (3) Tumours that had a relapse of the disease with distant metastases more than 6 months later (metachronous).

Primary CCRCCs with synchronous metastasis showed the highest FAP positivity (Figure 3E). This result was significant both at the centre and at the infiltrating front of the tumour when compared to not metastatic tissues. Primary CCRCCs with metachronous metastases showed also higher rate of FAP positivity than non-metastatic ones, and this difference was significant at the tumour centre.

Plasma analyses of sFAP showed also significant differences between these three groups of patients (Figure 3F). However, in this case, patients with synchronous metastasis showed significantly lower sFAP levels.

### 2.4. FAP Expression and Plasma Levels in Terms of the Cancer-Specific Survival (CSS) of CCRCC Patients

**Figure 4:**
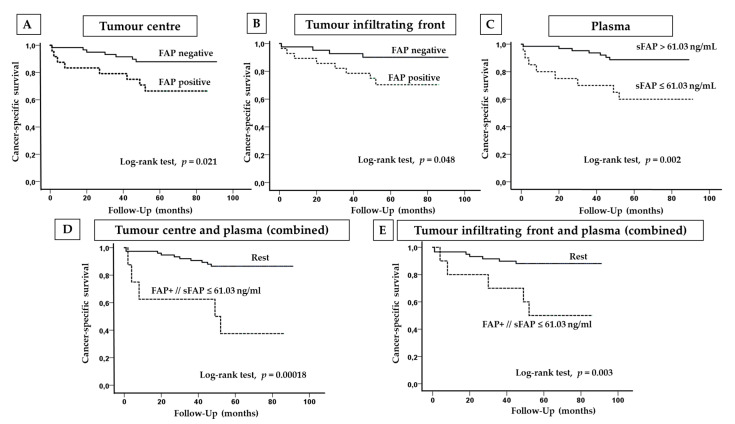
Immunohistochemical FAP and plasma sFAP levels according to CCRCC patients’ cancer-specific survival (CSS). Kaplan–Meier curves and univariate log-rank test showed that FAP expression at tumour centre (**A**) and border (**B**) is associated to worse CSS. CCRCC patients with lower sFAP levels than 61.03 ng/mL had worse CSS (**C**). Simultaneous expression of FAP at tumour centre (**D**) or border (**E**) and lower plasma FAP levels than 61.03 ng/mL are associated to worse CSS.

FAP positive immunostaining at the tumour centre and at the infiltrating front was significantly associated with worse 5-year cancer-specific survival (CSS) of CCRCC patients (Figure 4A,B).

Cut-off values of sFAP for CSS analyses were obtained by a Classification and Regression Tree (CRT) (Figure S2). A plasma sFAP value of 61.03 ng/mL determined two nodes with significant differences in the percentage of deceased patients, 40% vs. 10% (*p* = 0.03). Kaplan–Meier curves showed that CCRCC patients with low soluble FAP levels (≤61.03 ng/mL) presented worse CSS than patients with sFAP levels above this cut-off (Figure 4C).

Since tumour and plasma were obtained from the same patient, we also performed Kaplan–Meier curves by combining data of tissue FAP expression and plasma sFAP levels. Thus, two groups were made: (1) FAP-positive cases, at the centre or at the infiltrating front of tumours, with sFAP levels equal or lower than 61.03 ng/mL; and (2) the rest of possible combinations (FAP+/sFAP > 61.03 ng/mL; FAP-/sFAP ≤ 61.03 ng/mL; and FAP-/sFAP > 61.03 ng/mL).

CCRCC patients with tumour FAP positivity and sFAP ≤ 61.03 ng/mL had worse 5-year CSS than patients with the rest of the combinations (Figure 4D,E). Statistically significant results were found both at the centre and at the infiltrating front.

We performed ROC curves to determine the sensitivity and specificity of cut-off values of tissue and plasma FAP to predict 5-year CSS (Table S2). The sensitivity of FAP at the tumour centre and at tumour front was of 53.3 and 66.7% respectively, whereas the specificity was of 76.5% and 64.9%. Plasma sFAP showed 53.3% of sensitivity and 82.4% of specificity. The combination of FAP expression at the tumour centre with sFAP levels (cut-off value = 61.03 ng/mL) showed low sensitivity (33.3%) but high specificity (95.6%). Similar results were obtained for the combination between FAP at the tumour front and sFAP (sensitivity = 41.7%; specificity = 91.2%).

Multivariate Cox regression analyses were performed to know whether FAP expression in tissue, plasma FAP levels, or the combination of both isoforms are independent prognostic factors for 5-year CSS (Table 2). The analysis revealed that plasma sFAP with this cut-off value was an independent prognostic factor for CSS, together with local invasion (pT) and metastasis.

## 3. Discussion

The expression of FAP in the cytoplasmic membrane is a hallmark of fibroblast activation in malignant tumours [10]. This serine peptidase is involved in several phenomena that enable cancer cells to reach invasive properties [26]. The increased expression of FAP at the invasive front of tumours may contribute to this invasive behaviour [20,27]. The relationship between FAP expression and poor clinical outcome of cancer patients is quite well known [19]. In this sense, we demonstrated that FAP expression in CCRCC is associated to tumour aggressiveness and worse survival and suggested that this protein can be a good candidate as prognostic biomarker for this cancer [21,22].

However, CCRCC is intrinsically heterogeneous [4], and the expression of several biomarkers can be also heterogeneous in this tumour [4,8,12], which is a fact that could limit the usefulness of biomarkers in the clinic routine. We studied FAP expression at the centre and the infiltrating front of RCCs, and the presence of this protein was quite homogeneous in both areas. We validated previous results [21,22] in a new series of CCRCCs, and the association between FAP positivity and worse prognosis was confirmed, regardless of the area of the tumour in which the protein was expressed. This result play in favour of a potential use of FAP as an immunohistochemical biomarker of renal cancer prognosis. Furthermore, it suggests that the expression of this protein in CAFs does not have a specific role in the achievement of the infiltrating capacity of renal cancer cells in the tumour front.

The IHC analysis of FAP did not reveal any significant difference among CCRCC, PRCC, and ChRCC. However, the unique benign tumour analysed in this study, RO, was negative for this protein. We expected this result because FAP is a marker of fibroblasts of tumour tissues with an infiltrating nature [19]. The significant difference between malignant tumours and RO could be of clinical interest in the differential diagnosis between ChRCC and RO. These tumours arise from the intercalated cells of the distal nephron and are considered extremes of the same morphological spectrum [3]. The difficulty for the histopathological differentiation of these entities still persists and has important consequences in the management of patients [3]. Until now, the efforts in the research of new biomarkers have been focussed mainly on the analysis of tumour cells [3]. However, our results demonstrate that the IHC analysis of CAF-related markers could be a complementary alternative. A limitation of our study was that the series was small and that FAP appeared only in less than half of the ChRCCs. Although the specificity was the highest, the sensitivity of the IHC determination of FAP for the differential diagnosis of ChRCC and RO was low. Therefore, new IHC analyses combining FAP with other CAF-related proteins in larger tumour series are necessary to gain in sensitivity and to corroborate the usefulness of these biomarkers as new diagnostic tools.

Analysis of plasma from CCRCC patients revealed interesting findings. Thus, low levels of plasma sFAP in CCRCC patients were found to be significantly associated with tumours larger than 7 cm, a high histological grade, lymph node invasion, and the onset of synchronous distant metastases. Moreover, low sFAP levels were found to be independently associated with a worse 5-year CSS; in addition, the concurrence of these low levels and tissue FAP positivity also predicted the poor survival of CCRCC patients. These findings indicate that FAP analysis, either of tissue or liquid biopsy, can be used to predict the prognosis of the disease.

However, these data already highlight a contribution to the controversial debate regarding the origin of the soluble isoform of FAP and its variations in the plasma of cancer patients [17,18,20,28]. Thus, FAP is highly expressed in fibroblasts found in chronic inflammation and fibrotic lesions, as well as in cancer tissues [19,29]. It follows that higher levels of soluble FAP would be expected in the plasma from cancer patients. However, lower levels of sFAP were found in our patients with renal tumours in comparison to control subjects. Furthermore, plasma sFAP levels from patients with FAP-negative RCCs were very similar to those in patients with FAP-positive tumours.

Similar paradoxically reduced levels of sFAP have been reported in various studies to be associated with the cancer state and worse survival of patients [17,20,28]. Thus, it should be considered that sFAP may not be produced by the tumour tissues. Indeed, other physiological sources of sFAP have recently been reported including skeletal muscle, liver, and bone marrow [18]. Thus, it is conceivable that lower sFAP levels in pathological conditions may be indicative of a systemic reaction to a developing tumour [17,18,20,28]. Another possible pathophysiological mechanism related to decreases of sFAP levels could be associated to the characteristic weight loss of RCC patients with metastatic status [30]. In fact, a recent study described decreased plasma levels of this protein in obese adults with diet-induced weight loss [31]. Although this study highlighted that the subcutaneous adipose tissue was not the contributor of these changes in sFAP levels, it should not be discarded that one of the mechanism under the decreases of sFAP in cancer patients [17,20,28] could be related to this phenomenon. Further analyses will be necessary to validate these hypotheses, and it will be crucial to identify the origin of plasma sFAP before it can be considered to be a reliable biomarker for use with liquid biopsies of renal cancer patients.

The therapeutic implications of our findings support that CAF-directed therapy could be designed to prevent CAF activation or to redirect CAFs back into a normal phenotype. In particular, FAP has been the focus of different targeted therapies including low molecular weight inhibitors, FAP-mediated activation of prodrugs, and immune-based therapies [32]. Some of these stromal targets are being evaluated in pancreatic cancer, either by depleting CAFs using CAF-related cell-surface markers, such as α-SMA or FAP, reprogramming CAFs into quiescent fibroblasts, or targeting interactions between CAFs and their surrounding microenvironment [33]. Additionally in pancreatic cancer, targeting the chemokine (C-X-C motif) ligand 12 (CXCL12) from FAP-expressing carcinoma-associated fibroblasts synergises with anti-PD-L1 immunotherapy [34], and this is a very interesting route to explore the therapeutic potential of FAP expression in CCRCC, particularly in patients non-responding to immunological checkpoints antagonists. Future studies will help to elucidate all these therapeutic implications of FAP in RCC.

## 4. Materials and Methods

The present study including all its experiments comply with current Spanish and European Union legal regulations. The Basque Biobank for Research-OEHUN (www.biobancovasco.org) was the source of samples and the data from patients employed could possibly be used for research purposes. Each patient signed a specific document that had been approved by the Ethical and Scientific Committees of the Basque Country Public Health System (Osakidetza) (PI+CES-BIOEF 2018-04).

### 4.1. Patients

Tumour tissues and plasmas from 128 RCC patients surgically treated at Basurto University Hospital between 2012 and 2016 were collected for the study. The tumour centre and infiltrating front of RCC samples were included in tissue microarrays (TMAs) for further immunohistochemical analyses. Clinical follow-up of patients extended from RCC diagnostic data to 31 December 2019. Table 3 summarises the clinical and histological characteristics of patients with renal tumours.

Plasma from 46 healthy volunteers with no clinical history of neoplastic diseases was used as control sample (male/female 28/18, age 55.8/61.8 years).

American Joint Committee on Cancer (AJCC) [35] and Furhman’s [36] methods were applied to assign stage and grade, respectively.

### 4.2. Immunohistochemistry

FAP in formalin-fixed and paraffin-embedded tissues was immunodetected using FAP-specific antibodies (1:70 dilution, Ab53066 Abcam, lot: GR 3189477-2) as described previously [20,21,22,37]. Immunostaining was performed using an automatic immunostainer following standard procedures (DakoAutostainer Plus, Dako-Agilent). Briefly, antigens were retrieved by incubation for 20 min in low pH buffer (K8005, Dako, Santa Clara, CA. USA) at 95 °C. Incubation with the rabbit primary antibody was carried out for 50 min at room temperature. Following washing of the primary antibody, samples were incubated with an HRP (horseradish peroxidase)-labeled secondary anti-rabbit serum (K8021, Dako City, Santa Clara, CA. USA) for 20 min. An HRP enzyme labelled polymer (SM802, Dako) was employed using the EnVision-Flex detection system. The presence of HRP was visualised with diaminobenzidine (DAB) solution (DM827, Dako Santa Clara, CA. USA) and finally, sections were haematoxylin counterstained (K8008, Dako Santa Clara, CA. USA).

All these procedures were carried out attending to instructions given from the trading house and based on recent research studies that showed sharp membranous/cytoplasmic staining in stromal cells of tumour tissues [19,38]. This staining procedure and the same antibody were previously applied by this research group in renal [21,22], colorectal [20], and urothelial cancers [37].

Considering the extreme intertumour heterogeneity detected in 2.5 mm samples of the TMAs, pathologists determined that the score “no/low/medium/high” was difficult and not reproducible because the amount of stromal fibroblasts stained was very variable in quantity, so the score evaluation was not robust enough. Therefore, we documented the presence (+) or absence (−) of FAP immunolabel in stromal fibroblasts adjacent to neoplastic nests, using a Nikon Eclipse 80i microscope (Tokyo, Japan). All specimens were independently evaluated by two observers; in the event of discrepancies, samples were re-evaluated to arrive at a final conclusion.

### 4.3. ELISA Assays

Levels of soluble FAP were evaluated using the sFAPDuoSet ELISA kit (R&D Systems, DY3715 McKinley Place NE, MN. USA) [39,40]. Standards (100 µL), together with plasma samples (1/100 dilution) and reagent blank were plated into a 96-well plate and incubated overnight at 4 °C. Following four washings, labelled FAP antibody (100 µL) was added to each well (except to the blank) and incubation was allowed to proceed for 1 h at 4 °C. Subsequently, 5 washes were carried out before the addition of chromogen (100 µL), which was incubated at room temperature for 30 min. Stop solution (100 µL) was added to each well, and absorbance was measured at 450 nm using the reagent blank as the zero standard.

### 4.4. Statistical Analysis

SPSS^®^ 24.0 software was used for the statistical analysis.

In order to assess if data obtained from tissue and plasma samples followed a normal distribution, we applied a Kolmogorov–Smirnov test. Based on this information, data were further analysed using parametric or non-parametric tests.

The correlation between FAP expression in tumours and sFAP plasma levels and patient age and gender was evaluated using Spearman Rho tests. Comparison of plasmatic FAP levels between two groups or more (respectively) was carried out using Mann–Whitney (Mann-U) and Kruskal–Wallis tests. To analyse categorical FAP expression (negative/positive) in RCC tissues and to test the association of differences with pathological variables, we employed the Chi-square (χ2) test.

Cancer-specific survival (CSS) analyses were performed following the establishing of groups by cut-off points, following different methods: (I) for tissue analyses, cut-off points were based on the categorical expression of FAP (negative vs. positive); (II) for plasma analyses, cut-off values of sFAP were obtained with classification and regression tree (CRT) method. ROC curves were performed to determine the sensitivity and specificity of these cut-offs values. In order to evaluate the CSS of CCRCC patients, Kaplan–Meier curves and log-rank tests were carried out. Finally, to evaluate the independent effects of FAP expression and sFAP levels and clinical and pathological variables on CSS, we used multivariate analyses (Cox regression model).

## 5. Conclusions

The findings that we report here provide evidence for the potential usefulness of FAP and its soluble isoform as a biomarker of CCRCC progression. In addition, our data indicate that CAF-related proteins are potential immunohistochemical biomarkers for the differential diagnosis of ChRCC and RO. It is hoped that future studies will contribute to a more fine-grained understanding of the role of FAP in intercellular communication within the EMT of renal tumours. The design and implementation of more effective CAF-targeted therapies may well be on the horizon.

## Figures and Tables

**Table 1:** Plasma soluble FAP (sFAP) levels in RCC patients depending on FAP expression in tumour tissues. Data were analysed by Mann-Whitney test.

	FAP at the Tumour Centre			FAP at the Infiltrating Front		
Plasma sFAP (ng/mL)	Negative	Positive	*p*=	Negative	Positive	*p*=
CCRCC	89.1 ± 4.4	90.5 ± 8.6	0.69	90.4 ± 5.5	90.9 ± 8.4	0.64
PRCC	95.9 ± 11.2	94.1 ± 4.9	0.87	89.9 ± 11.1	92.9 ± 4.4	0.51
ChRCC	57.3 ± 11.8	86.74 ± 23.8	0.29	53.4 ± 9.3	92.1 ± 22.1	0.16

**Table 2:** Predictive model (Cox regression) for 5-year cancer-specific survival (CSS) prediction in CCRCC patients. Selected independent variables were FAP expression in the primary tumour’s centre and infiltrating front (**A**); plasma sFAP (≤ or > 61.03 ng/mL) (**B**); combination of FAP expression in tumour and plasma sFAP (FAP+/sFAP ≤ 61.03 ng/mL vs. rest of combinations) (**C**); and pathological variables such as Fuhrman grade, local invasion (pT), lymph node (N), and distant (M) metastases. ExpB with confidence interval (CI, inferior and superior) is also included. Statistically significant values are highlighted in bold.

A									
		Tumour Centre				Infiltrating Front			
5-Year CSS	Variables	*p*=	ExpB	Inf	Sup	*p*=	ExpB	Inf	Sup
Multiple Cox Regression	FAP	0.26	0.51	0.15	1.65	0.41	0.53	0.12	2.35
	Grade	0.2	2.92	0.57	15	0.56	1.93	0.21	18.17
	pT	**0.03**	2.12	1.08	4.17	0.08	2.01	0.92	4.4
	N	0.11	2.75	0.8	9.41	0.11	3.23	0.76	13.66
	M	**0.001**	35.76	4.3	297.7	**0.004**	29.58	2.97	294.8
Final Step of Wald Method	pT	0.052	1.95	0.99	3.81	**0.039**	2.15	1.04	4.46
	N	0.07	3.04	0.91	10.1	-			
	M	**0.001**	33.24	4.21	262.4	**0.001**	29.94	3.78	237.4
**B**									
		**Plasma**							
**5-Year CSS**	**Variables**	***p*** **=**	**ExpB**	**Inf**	**Sup**				
Multiple Cox Regression	sFAP	0.079	4.67	0.83	26.18				
	Grade	0.5	1.75	0.34	9				
	pT	**0.01**	2.71	1.22	6.01				
	N	0.7	0.7	0.11	4.45				
	M	**0.002**	28.47	3.39	238.8				
Final Step of Wald Method	sFAP	**0.011**	4.24	1.39	12.95				
	pT	**0.007**	2.51	1.29	4.89				
	M	**0.001**	32.5	4.13	256.2				
**C**									
		**Tumour Centre**				**Infiltrating Front**			
**5-Year CSS**		***p* =**	**ExpB**	**Inf**	**Sup**	***p* =**	**ExpB**	**Inf**	**Sup**
Multiple Cox Regression	FAP/sFAP	0.98	0.98	0.2	4.87	0.83	1.28	0.13	12.1
	Grade	0.32	2.24	0.46	10.98	0.6	1.84	0.19	18.1
	pT	**0.048**	1.97	1.01	3.85	0.12	1.96	0.84	4.61
	N	0.27	2.55	0.48	13.43	0.55	2.08	0.19	22.6
	M	**0.002**	27.46	3.37	223.5	**0.007**	20.81	2.34	185.3
Final Step of Wald Method	pT	0.52	1.95	0.99	3.81	**0.039**	2.15	1.04	4.45
	N	0.07	3.04	0.91	10.1	-			
	M	**0.001**	33.24	4.21	262.4	**0.001**	29.9	3.78	237.4

**Table 3:** Clinical and pathological parameters of CCRCC patients, and gender and age of patients with PRCC, ChRCC and RO.

Tumour Subtype	CCRCC (*n* = 89)	PRCC (*n* = 21)	ChRCC (*n* = 8)	RO (*n* = 8)
Age (range)	61.4 (36–82)	54.0 (26–80)	64.6 (47–74)	63.5 (40–84)
Sex (Male/Female)	60/29	17/4	7/1	3/7
Follow-up (months)	59.9 (1–91)			
Survival				
Alive	68			
Dead of disease	15			
Dead by other causes	6			
Diameter				
≤4 cm	28			
>4 to 7cm	39			
>7 cm	22			
Fuhrman grade				
G1	3			
G2	46			
G3	30			
G4	10			
Local invasion (pT)				
pT1	59			
pT2	12			
pT3	16			
pT4	2			
Lymph node invasion (*N*)				
No	83			
Yes	6			
Distant metastasis (M)				
No	66			
Synchronous	10			
Metachronous	13			

## Supplementary Materials

The following are available online at https://www.mdpi.com/2072-6694/12/11/3393/s1, Figure S1: FAP immunostaining in centre (A) and border (B) of CCRCC tissues in terms of local invasion (pT). FAP-positive cases at the centre of the tumour were significantly higher in non-organ-confined and pT2 tumours than in pT1 ones. At the infiltrating front of these non-organ-confined tumours, FAP positive cases almost duplicated the expression of pT1 tumours, although it did not reach statistical significance, and pT2 tumours had higher FAP expression than pT1 ones. Figure S2: Classification and Regression Tree (CRT). A plasma FAP (sFAP) value of 61.03 ng/mL determined two nodes with significant differences in the percentage of alive patients (*p* = 0.034). Table S1: Correlation between age, sex, tissue FAP expression and soluble FAP levels in CCRCC, PRCC, ChRCC, and RO patients (Spearman Rho test). Table S2: ROC Curves. Sensitivity and specificity of tissue and plasma FAP to predict cancer-specific survival (CSS) of CCRCC patients (*n* = 89). Sensitivity and specificity of tissue FAP to determine the potential of FAP staining in tumour tissues for the differential diagnosis between ChRCC (*n* = 8) and RO (*n* = 10). *AUC*: Area Under the Curve.
