# Supplementary material for: Altered Tissue and Plasma Levels of Fibroblast Activation Protein-α (FAP) in Renal Tumours
Source: Cancers (Basel). 2020 Nov 16;12(11):3393. doi: 10.3390/cancers12113393 (PMC7696212; doi:10.3390/cancers12113393)
Supplement: Supplementary file 1 [file cancers-12-03393-s001.pdf]

Altered Tissue and Plasma Levels of Fibroblast Activation Protein-α (FAP) in Renal Tumours

Jon Danel Solano-Iturri, Peio Errarte, María C. Etxezarraga, Enrique Echevarria, Javier Angulo, José I. López and Gorka Larrinaga

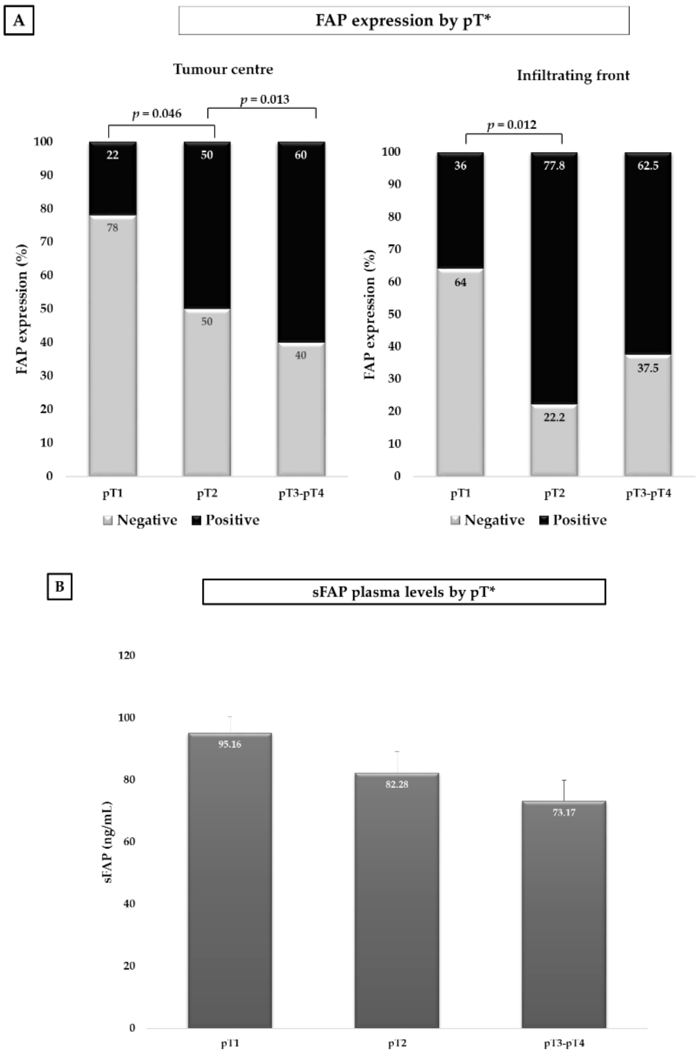

Figure S1. FAP immunostaining in centre (**A**) and border (**B**) of CCRCC tissues in terms of local invasion (pT). FAP positive cases at the centre of the tumour were significantly higher in non-organ-confined and pT2 tumours than in pT1 ones. At the infiltrating front of these non-organ-confined tumours, FAP positive cases almost duplicated the expression of pT1 tumours, although it did not reach statistical significance, and pT2 tumours had higher FAP expression than pT1 ones.

Classification and Regression Tree (CRT) for soluble FAP and CCRCC patients' cancer-specific survival (CSS)

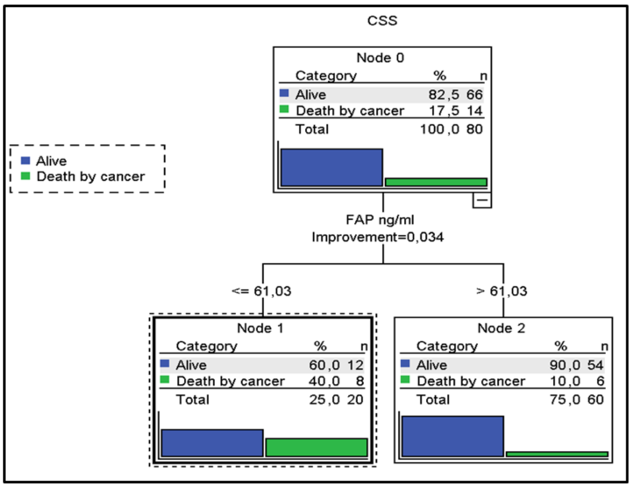

Figure S2. Classification and Regression Tree (CRT). A plasma FAP (sFAP) value of 61.03 ng/mL determined two nodes with significant differences in the percentage of alive patients ($p = 0.034$).

Table S1. Correlation between age, sex, tissue FAP expression and soluble FAP levels in CCRCC, PRCC, ChRCC and RO patients (Spearman Rho test).

		**Primary tumour**		**Plasma**
**CCRCC**		**FAPc**	**FAPb**	**sFAP**
**Sex**	r =	-0.130	-0.098	-0.142
	p =	0.22	0.41	0.192
**Age**	r =	0.051	0.103	0.021
	p =	0.64	0.38	0.84
**PRCC**				
**Sex**	r =	-0.231	-0.077	-0.160
	p =	0.34	0.75	0.49
**Age**	r =	0.019	-0.039	0.098
	p =	0.94	0.87	0.67
**ChRCC**				
**Sex**	r =	-0.354	-0.354	-0.577
	p =	0.44	0.44	0.13
**Age**	r =	-0.577	-0.144	-0.381
	p =	0.17	0.76	0.352
**RO** (*)				
**Sex**	r =	-	-	-0.114
	p =	-	-	0.75
**Age**	r =	-	-	0.127
	p =	-	-	0.71

(*) FAP expression in RO tissues was negative in all cases. For this reason, the correlation test was performed only between age, sex and plasma sFAP levels. FAPc = FAP expression in the centre of the tumour. FAPb = FAP in tumour border.

Table S2. ROC Curves. Sensitivity and specificity of tissue and plasma FAP to predict cancer-specific survival (CSS) of CCRCC patients (n = 89). Sensitivity and specificity of tissue FAP to determine the potential of FAP staining in tumour tissues for the differential diagnosis between ChRCC (n = 8) and RO (n = 10). *AUC*: Area Under the Curve.

	ROC curves for CSS		
	**AUC**	**Sensitivity**	**Specificity**
**FAP Centre (-/+)**	0,649	53.3%	76.5%
**FAP Front (-/+)**	0,658	66.7%	64.9%
**sFAP (≤ or > 61,03 ng/mL)**	0,678	53.3%	82.4%
**FAP Centre / sFAP**	0,645	33.3%	95.6%
**FAP Front / sFAP**	0,664	41.7%	91.2%
	**ROC curves for differential diagnosis between ChRCC and RO**		
	**AUC**	**Sensitivity**	**Specificity**
**FAP Centre (-/+)**	0.714	42.9%	100%
**FAP Front (-/+)**	0.714	42.9%	100%

© 2020 by the authors. Licensee MDPI, Basel, Switzerland. This article is an open access article distributed under the terms and conditions of the Creative Commons Attribution (CC BY) license (http://creativecommons.org/licenses/by/4.0/).
